# Innate lymphocytes: Role in alcohol-induced immune dysfunction

**DOI:** 10.3389/fimmu.2022.934617

**Published:** 2022-08-29

**Authors:** Karla Ruiz-Cortes, Daniel N. Villageliu, Derrick R. Samuelson

**Affiliations:** College of Medicine, Department of Internal Medicine, Division of Pulmonary, Critical Care and Sleep, University of Nebraska Medical Center, Omaha, NE, United States

**Keywords:** pneumonia, bacteria, alcohol, innate immunity, innate lymphocytes

## Abstract

Alcohol use is known to alter the function of both innate and adaptive immune cells, such as neutrophils, macrophages, B cells, and T cells. Immune dysfunction has been associated with alcohol-induced end-organ damage. The role of innate lymphocytes in alcohol-associated pathogenesis has become a focus of research, as liver-resident natural killer (NK) cells were found to play an important role in alcohol-associated liver damage pathogenesis. Innate lymphocytes play a critical role in immunity and homeostasis; they are necessary for an optimal host response against insults including infections and cancer. However, the role of innate lymphocytes, including NK cells, natural killer T (NKT) cells, mucosal associated invariant T (MAIT) cells, gamma delta T cells, and innate lymphoid cells (ILCs) type 1–3, remains ill-defined in the context of alcohol-induced end-organ damage. Innate-like B lymphocytes including marginal zone B cells and B-1 cells have also been identified; however, this review will address the effects of alcohol misuse on innate T lymphocytes, as well as the consequences of innate T-lymphocyte dysfunction on alcohol-induced tissue damage.

## Introduction

**Figure 1:**
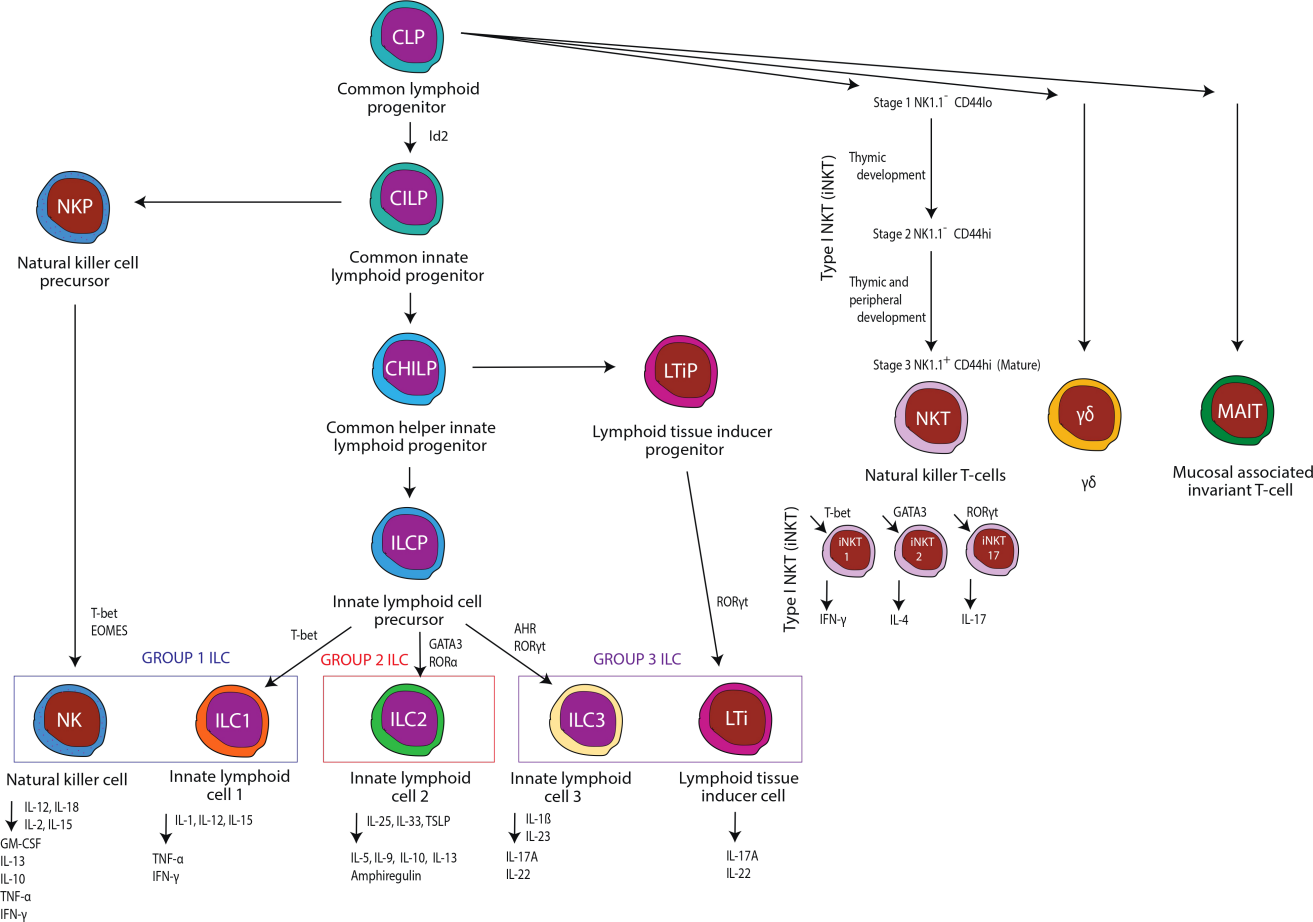
An overview of innate and innate-like immune cells examined in this review.

A complete immune response requires optimal and timely responses from both tissue-resident and circulating immune cell populations. Innate immune cells are often found in peripheral tissues and respond to various infectious challenges, cancers, and allergens through the expression of toll-like receptors (TLRs) and limit tissue injury *via* the production of a wide variety of TLR-dependent effectors. However, there is a growing appreciation and understanding of the scope and diversity of tissue-resident lymphocytes in peripheral organs, which suggests that myeloid cells may not be the primary, or only, immune response prior to the initiation of classical adaptive immunity (1). Innate lymphocytes are broken into two distinct groups: innate lymphoid cells (ILCs) and innate-like T lymphocytes. Innate lymphocytes have been shown to mediate normal host immune responses to various infectious challenges, cancers, and allergens, as well as provide immune regulatory and modulatory effects. Currently, ILCs are classified into five subsets within three major groups: natural killer (NK) cells, ILC1 (group 1), ILC2 (group 2), and ILC3 and LTi cells (group 3) (1). Innate-like T lymphocytes or unconventional T cells are typically classified as gamma delta (γδ) T cells, mucosal associated invariant T cells (MAIT), natural killer T cells (NKT), and invariant natural killer T cells (iNKT) and, similar to ILCs, mediate both immune responses and homeostasis (1). This review will address our current knowledge regarding the effects of alcohol misuse on these innate and innate-like lymphocytes (**Figure 1**), as well as the consequences of innate lymphocyte dysfunction on alcohol-induced end-organ damage.

## Innate lymphocytes: Role and function in immune homeostasis

### Innate lymphoid cells

ILCs utilize a variety of germline-encoded activating and inhibitory receptors, as opposed to conventional lymphocytes which express rearranged antigen receptors (2). ILCs are primarily located at epithelial barrier surfaces (i.e., intestine, lung, and skin) but can also be identified in lymphoid and other non-lymphoid tissues (3). ILCs are often classified based on their expression of transcription factors, cell surface markers, cytokines, and effector molecules. Following tissue injury due to infection or inflammation, as well as perturbation to the intestinal commensal microbiota, ILCs produce both proinflammatory and regulatory cytokines to combat the tissue insult (1, 3, 4). Nearly every organ has associated tissue-specific ILCs, which attests to their ability to support many critical functions necessary for immune homeostasis.

#### Group 1 ILCs

NK cells and type 1 ILCs (ILC1), which are defined based on the secretion of interferon (IFN)-γ, are the prototypical group 1 ILC. Group 1 ILCs are highly responsive to interleukin IL-15, IL-18, and IL-12 and are typically characterized by the expression of the surface receptors NKp46 and NK1.1 (mice) or CD56 (humans) (5). ILC1s are often considered to be more tissue specific/resident than NK cells and express higher levels of CD103, CD49a, and CD69, all of which are considered markers of tissue residency (1). Conversely, NK cells typically express surface markers that facilitate circulation, such as CCR7, S1PR, and CD62L (6). Likewise, NK cells are more cytolytic than ILC1s, as they have a higher expression of both perforin and granzymes (7). However, ILC1s also have the potential to be cytolytic *via* the production of the tumor necrosis factor-related apoptosis-inducing ligand. Innate lymphoid cell precursor (ILCP) differentiation into ILC1 requires the transcription factors T-bet and Hobit (8, 9); conversely, differentiation of precursor NK cells into mature NK cells is dependent on the transcription factors Eomes and T-bet (10). While these transcription factors are widely accepted for cellular development in rodents, the transcriptional profile for human ILC1s and NK-cell development is less defined. Eome expression is found in intestinal intraepithelial ILC1s (11) but, until recently, was not believed to contribute to liver-resident NK cells (12). Interestingly, however, it has been shown in humans that a liver-resident Eomes^hi^ NK-cell population does exist (13). In fact, it appears that Eomes expression in humans is a factor for NK retention. Cuff et al. examined liver transplants from donors which were HLA mismatched (HLA-A2 or HLA-A3 mismatches). This allowed them to distinguish between donor liver–derived and recipient-derived leucocytes *via* antibody staining for the specific donor-recipient HLA mismatch. They found that Eomes^lo^ NK cells circulate freely whereas Eomes^hi^ NK cells were only observed in the liver and not found in blood samples. Cuff et al. went to further establish that liver NK-cell replenishment from the circulation can occur, possibly *via* Eomes^lo^ NK cells being induced to upregulate Eomes expression. These data suggest that Eomes^hi^ expression may be a characteristic of mature liver NK cells; however, the role of Eomes expression in NK development remains clouded. For example, some authors have described Eomes expression as part of NK-cell development but have also argued that the Eomes^hi^ state is associated with immature NK cells. They argue instead that mature NK cells are more associated with an abundance of T-bet (14). Whatever the case may be, NK cells and ILC1s are best known for their critical role in the normal immune responses to viral infection through secretion of IFN-γ.

#### Group 2 ILCs

The secretion of the classical type-2 cytokines amphiregulin, IL-13, IL-9, and IL-5 in response to IL-33, IL-25, and TSLP secreted by parenchymal cells is one key defining feature of group 2 ILCs (3, 4, 15). ILC2s are also classically defined by the expression of CRTH2, KLRG1, ST2, and CD25 (16, 17). Interestingly, the expression of CD44 and CD161 on ILC2 seems to differ between mice and humans, as mouse ILC2s are CD44+ CD161-, while human ILC2s are CD44- CD161+ (18). Differentiation from ILCPs into mature ILC2s depends on the transcription factors GATA3 (also required for effector function), RORα, and TCF-1 (19–23). Recently, ILC2s have been sub-characterized *via* their ability to respond to IL-33 (natural ILC2s) and IL-25 (inflammatory ILC2s), or their ability to secrete IL-10 (ILC2_10_) (24–26). The ILC2-mediated secretion of IL-13 and amphiregulin is critical for the repair of tissue damage following helminth or viral infections. IL-13 is also important for host-mediated removal of helminths.

#### Group 3 ILCs

Group 3 ILCs (innate counterparts of Th17 T cells) typically produce IL-22 and IL-17A following activation by IL-1β and IL-23. Furthermore, ILC3 can also secrete TNF-α and GM-CSF in response to stimulation (27, 28). ILC3 development from ILCPs is primarily driven by three key transcription factors: 1) aryl hydrocarbon receptor (AhR), 2) promyelocytic leukemia zinc finger (PLZF), and 3) retinoid-related orphan receptor γt (RORγt) (29–31). Similarly, lymphoid tissue inducer (LTi) cells, a unique subtype of ILC3s, also require RORγt for differentiation and similarly produce the cytokines IL-22 and IL-17; however, PLZF in not necessary for development (30). Similarly, ILC3s are classified by their expression of NKp46, CD127, c-Kit, and CCR6. However, in mice CCR6^+^NKp46^−^ LTi cells as well as CCR6^−^NKp46^−^ and CCR6^−^NKp46^+^ ILC3 cells have also been described. ILC3 through the production of IL-22 play an integral role in immune homeostasis, by stimulating antimicrobial peptide production by epithelial cells and goblet cell mucus secretion, both of which support barrier integrity. Additionally, the ILC3 secretion of IL-17 and GM-CSF promotes granulopoiesis, the production of neutrophil chemoattractant (32), as well as the generation and survival of myeloid cells, and tolerogenic T cells (27).

### Innate-like T lymphocytes

Alongside ILCs, innate-like T lymphocytes participate in host defense against tissue damage or pathogenic insult prior to the adaptive immune response. Unconventional T-cell subsets express restricted T-cell receptor (TCR) sequences. Consequently, unconventional T-cell stimulation occurs independent of the classical major histocompatibility complex (MHC) I and II-dependent presentation of microbial components and/or antigens (33). Like ILCs, the classification of unconventional T cells depends on cytokines, effector molecules, transcription factors, and surface markers (**Table 1**). Growing evidence supports an important role of unconventional T cells in the early immune response by providing an immediate cellular response and facilitating conventional T-cell responses (33).

**Table 1:** Innate-like immune cells: recognized surface markers, effectors, and transcription regulators.

Type of cell	Surface markers	Non-cytokine effectors	Key cytokines	Transcription factors	Citation
MAIT	*αβ* T‐cell receptor with a semi‐invariant TCR‐α chain (usually Vα7.2−Jα33) associated with TCR‐β chains *(Vβ2, Vβ13), CD161^high^, CD3^+^, CD8α^+^, MR1*	Perforin, granzyme B	TNF‐*α*, interleukin‐17, IFN‐*γ*, IL‐4, IL-22	RAR-related orphan receptor γt (RORγt), promyelocytic leukemia zinc finger protein (PLZF), and eomesodermin (EOMES)	(34)
ILC-1	NKp46/NCR1, CD56, CD122, NK1.1/CD161, CD49a, CD103, Integrin α1, CXCR6, CXCR3, CD103, CD69, and CD39, CD127/IL-7 receptor α		IFN‐γ	T-bet, Hobit	(1, 35)
ILC-2	CRTH2, KLRG1, ST2, CD25, variant CD44, and CD161 expression		IL-5, IL-9, IL-13, amphiregulin	GATA-3, ROR-α, TCF-1	(3, 4, 13, 14, 16, 20, 21)
ILC-3	Nkp44, CD127, c-Kit, and CCR6		IL-17A, IL-22, GM-CSF, TNF‐*α*	RORγt, lymphoid tissue inducer (Lti), aryl hydrocarbon receptor (AhR), and promyelocytic leukemia zinc finger	(27, 28, 36, 37)
NKT/iNKT	Invariant TCRα (iNKT), restricted TCRβ chains (iNKT), greater diversity of TCRα and TCRβ chains (type 2 NKT)	Perforin, granzyme B	TNF‐α, IFN‐γ, IL-17, IL-4		(33, 38–43)

#### Natural killer T cells and invariant natural killer T cells

Presentation of lipid antigens *via* CD1d is the major defining characteristic of NKT cells. NKT cells are classically subdivided into two distinct populations based on the expression of different TCR alpha chains (38–41). Type 1 NKT cells (iNKT cells) express an invariant TCRα chain and a limited TCRβ profile. Human iNKT cells typically express the TCRα chain Vα24-Jα18, while iNKT cells from mice expresses the TCRα chain Vα14-Jα18. NKT cells also possess cytotoxic capabilities due to the expression of perforin, CD95/CD95 L, and TNF (42). Conversely, type 2 NKT cells express an expanded TCRα and TCRβ profile (40, 43). Alpha-galactosylceramide (α-GalCer), a ceramide lipid attached to a polar galactose head, is a model CD1d antigen. iNKT cells react and expand rapidly in response to α-GalCer, which drives iNKT cells to a classical effector status characterized by the production of key immunoregulatory cytokines. Non-lipid antigen-specific responses in iNKT cells have also been reported; however, most iNKT cells drive innate and adaptive immune responses *via* tumor necrosis factor-α (TNF-α), IFN-γ, IL-17, and IL-4-mediated activation of antigen-presenting cells (APCs). Finally, iNKT cells can also be characterized based on specific cytokines and transcription factors unique to each subset. Specifically, iNKT cells are often subdivided into the following groups: 1) iNKT1 cells, which utilize T-bet and secrete IFN-γ, 2) iNKT2 cells, which are GATA-3 expressing and IL-4 secreting, and 3) iNKT17 cells, which are dependent of RORγt expression and produce IL-17 (44).

#### Mucosal associated invariant T cells

MAIT cells co-express a semi-invariant TCR alpha (α) and beta (β) chain and CD161. In humans, TCR Vα 7.2-Jα 33/12/20 and Vβ2/13 are the most common TCR αβ chains, while in mice TCR Vα 19-Jα 33 paired with Vβ6/20 classically defines MAIT cells (45, 46). Recognition of vitamin B (riboflavin and folic acid) metabolites *via* presentation through the highly conserved MHC class I-related molecule 1 (MR1) is widely viewed as one of the, if not the, main characteristic of MAIT cells. Upon stimulation, MAIT cells rapidly secrete IFN-γ, TNF-α, IL-2, and IL-17, as well as exhibit cytotoxic effects (45, 47–53). In addition to classical MAIT-cell activation *via* MR1 ligands, MAIT cells can be alternatively activated *via* IL-15, IL-18, and IL-12 without TCR engagement (54–56). RORγt and PLZF are the two key transcription factors for MAIT-cell development (57, 58). Interestingly, there appear to be tissue-specific populations of MAIT cells. For example, MAIT cells derived from the liver generally have higher levels of the tissue residency markers CD69 and CD103, as well as markers of cellular activation CD56, CD38, PD-1, and NKG2D, under normal physiological conditions (54, 55, 59). MAIT cells facilitate immune regulation both during normal physiological conditions and during pathogenic or antigenic insult.

#### Gamma delta T cells

γδ T cells represent a unique subset of unconventional T cells, as they exhibit characteristics of both innate and adaptive immune cells. For example, following insult γδ T cells respond rapidly and do not require clonal selection or TCR recognition-mediated differentiation (60). Additionally, γδ T cells can be further characterized into distinct populations based on their TCRδ chain expression. γδ T cells that express either the Vδ1 or Vδ2 TCR chain are the two most common populations. These γδ T-cell subsets seem to also display a tissue-specific tropic behavior. Vδ2+ γδ T cells are mainly located in the circulatory system, while Vδ1+ γδ T cells are primarily mucosal-associated (61). Vδ1+ γδ T cells are also long-lived cells that exhibit low levels of CD27 and high levels of granzyme B and CX3CR1 (62). In addition, Vδ1+ γδ T cells also retain their proliferative capacity and TCR sensitivity (62). Likewise, Vδ1+ γδ T cells secrete IFN-γ and TNF-α, as well as perforin and granzyme B following TCR or CD1d stimulation (60, 62, 63). Recently, Vδ3+ γδ T cells have been described and were found to be enriched with hepatic tissues. These cells are activated by CD1d stimulation, which drives the production of Th1, Th2, and Th17 cytokines. These cells were also demonstrated to exhibit cytotoxic activity (64). γδ T cells play an important role in host defense, especially within mucosal-associated tissues.

## Innate lymphocytes: The effects of alcohol misuse

**Figure 2:**
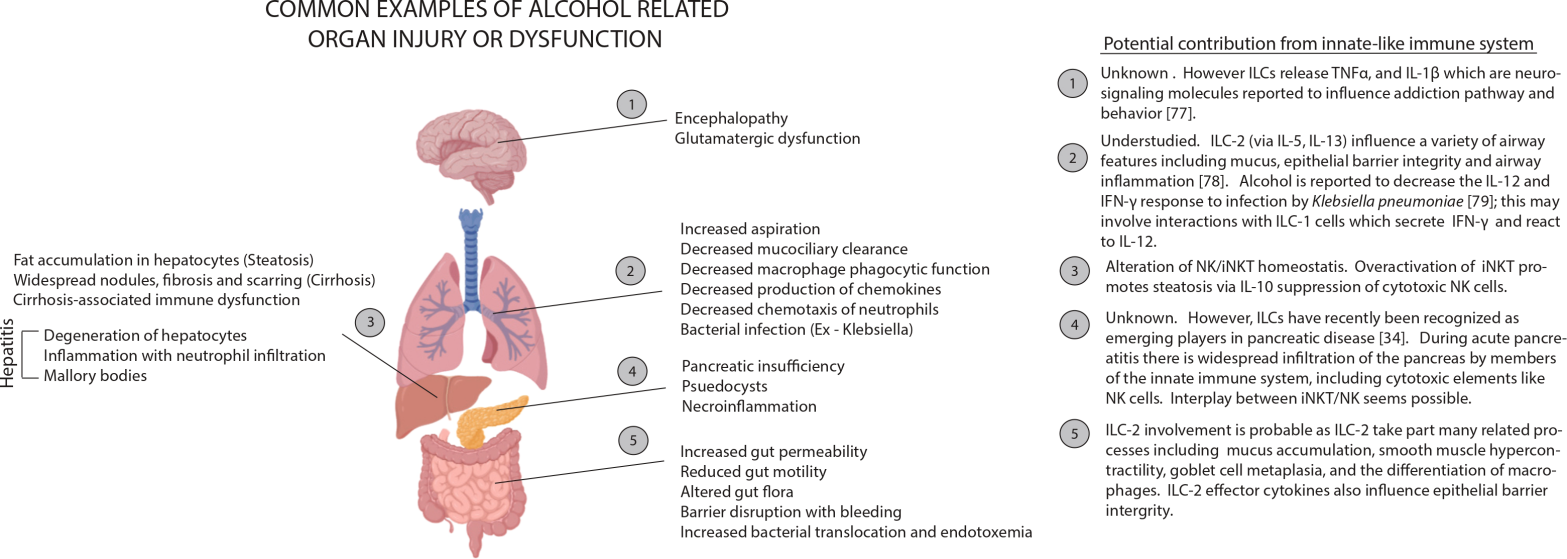
Examples of alcohol related organ injury with potential influences from the innate-like immune system. Many tissues rely on processes regulated by innate-like immune signaling to maintain homeostasis. Alcohol can perturb homeostasis by interfering with signal release (ex. decrease in IL-22 release by ILC-3 cells), by depleting or activating regulatory cells (ex. maturation of iNKT and inactivation of NK cells following alcohol exposure) or by interfering with effector cell function. Little has been rigorously established about how broad changes in the innate-like immune system result in tissue damage. However, we can make some informed inferences. Depletion of signals like IL-22 could facilitate injury in tissues like the lungs and small intestines (2, 5) because IL-22 is a fundamental mediator of inflammation, mucous production and tissue regeneration. During necrotic alcohol-associated tissue injuries (3, 4), there is often tissue infiltration by cytolytic elements including NK cells. In a healthy individual, the activity of these cytotoxic elements is kept in check by cytokine signaling by innate-like including iNKT cells. However, alcohol exposure can dysregulate this signaling and periods of hypo- and hyperactive cytolytic activity may result.

Alcohol use is known to alter the number and function of immune cells, such as macrophages, neutrophils, and T cells. This also appears to be true for innate lymphocytes (**Table 2**). Immune dysfunction has been associated with alcohol-induced end-organ damage (**Figure 2**). However, the role of innate lymphocytes remains ill-defined in the context of alcohol-induced end-organ damage. Below, we will highlight our current understanding of the effects of alcohol on innate lymphocyte populations.

**Table 2:** Innate-like immune cells: their function and known alcohol-related impairments.

Type of cell	General function	Alteration by alcohol	Citation
NK	Cytolytic effector lymphocytes which produce IFN-γ and act to control infection and tumor spread	Decreases the abundance and function of NK cells in the periphery, arrests development at the CD27+ CD11b+ stage, and impairs chemotaxis into inflamed/infected tissues. Alcohol also increases the number of IFN-γ-producing NK cells, while inhibiting the induction of perforin, granzyme A, and granzyme B following IL-2 stimulation.	(65–67)
ILC1	Production of proinflammatory and regulatory cytokines (particularly IFN-γ), maintenance of immune homeostasis. Usually tissue-specific residents.	ILC1 numbers are relatively unaffected by chronic alcohol administration, but ILC1 impairments in alcohol + infection murine models have been previously suggested. Understudied.	(65, 66, 68)
ILC2	Production of proinflammatory and regulatory cytokines (particularly type 2 cytokines). Critical to type 2 inflammation. Of particular importance to lung tissue homeostasis with influences on epithelial barrier integrity, mucus, and airway influence.	Likely dysregulated; however, we found no studies that evaluated the effects of alcohol on ILC2 cells in any tissue.	
ILC3	Production of proinflammatory and regulatory cytokines (particularly IL-22, IL-17A). In the gut, they serve as sentinels involved in maintaining homeostasis and tolerance to commensals while also functioning to prevent invasion by pathogens. The LTi subtype appears important for the long-term maintenance of memory CD4 T cells.	Ethanol impairs secretion of IL-22 from gut ILC3, which was correlated with alcohol-related changes in the composition of the intestinal microbiota and increased intestinal permeability.	(36, 69, 70)
NKT/iNKT	These cells are CD1d-restricted and react to lipid antigenic stimulation within minutes by secreting a wide variety of cytokines. This rapid response time makes these cells important in the early response to infection.	Alcohol increases proliferation and maturation of iNKT cells. These cells secrete IL-10 and IFN-γ which are both altered in alcohol use disorder.	(65, 71, 72)
γδ	Part of early rapid response to insult, these cells have characteristics of both innate and adaptive immune cells but do not require clonal selection or TCR recognition. Cytotoxic.	In a murine model, subsets of dermal γδ T cells (CD3hiVγ3+ and CD3intVγ3-) were diminished. Diminished IL-17 secretion.	(73, 74)
MAIT	MAIT cells react to key microbe-associated molecules (riboflavin) *via* a conserved TCR that recognizes MR1. Though they recognize a more restrictive subset of antigens than other conventional MHC-restricted T cells, their response is more rapid. These cells are a crucial part of the early infection response mounted in peripheral mucosal tissue like the lung and GI tract.	Chronically, alcohol depletes MAIT cells in the liver, GI, and lungs, as well as reduces their antibacterial activity. Alcohol also dysregulates cytokine production following infection in a tissue-specific manner.	(75, 76)

### Innate lymphoid cells

#### Group 1 ILCs

**Figure 3:**
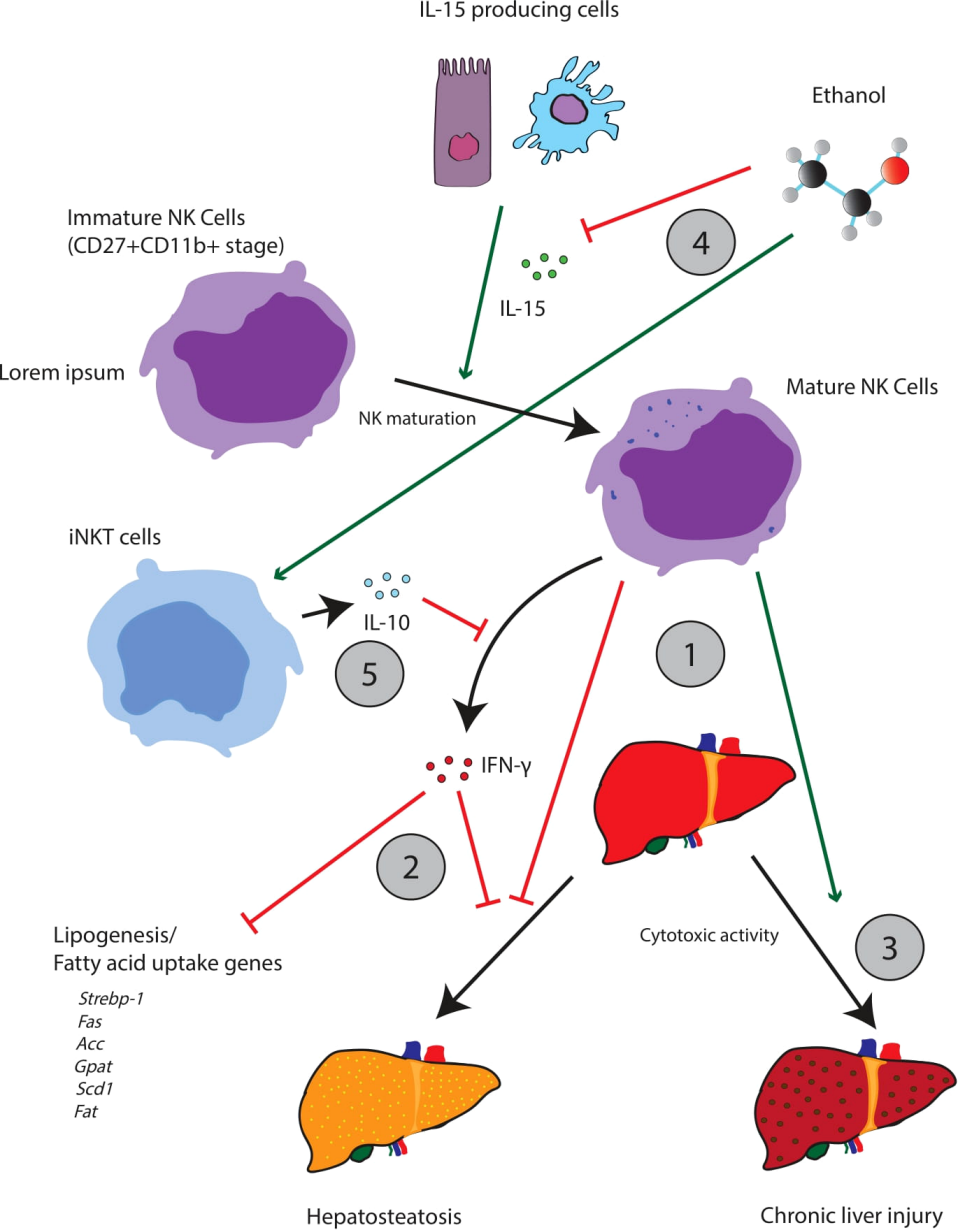
The interplay between iNKT cells and NK cells appears central to the pathogenesis of hepatic steatosis and other aspects of alcoholic liver disease. Mature NK cells appear to oppose hepatic steatosis, but also facilitate tissue injury through cytotoxic activity (1). Acutely, NK activity is thought to be beneficial; NK cells release IFN-γ which downregulates a variety of lipogenic and fatty uptake genes (2). NK cells can also promote beneficial remodeling and regeneration of the liver. Chronically however, over activation of NK cells may contribute to liver injury (3). Alcohol can perturb the iNKT/NK cell balance, favoring iNKT cell maturation while suppressing the maturation of NK cells (3). This change can be achieved through a variety of possible mechanisms. For example, ethanol impairs the release of IL-15 which promotes the maturation of NK cells (4). Ethanol can also promote the release of IL-10 from iNKT cells which will suppress NK activity (5).

Chronic alcohol consumption has been demonstrated to decrease the abundance and function of NK cells in the periphery (65). Zhang et al. demonstrated that following chronic alcohol exposure, NK cells are arrested in their development at the CD27^+^CD11b^+^ stage (**Figure 3**). Further, cytotoxic NK cells (cNK) appear to accumulate in the bone marrow with a corresponding drop in the number of cNK cells in tissues (i.e., the spleen, lung, liver, and lymph nodes). Given that cNK cells produce IFN-γ and the cytotoxic effector molecules perforin and granzyme B, it is likely that the impairment of cNK maturation alters the release of IFN-γ and cytotoxic effector molecules, which has further downstream effects/impairments in other components of the innate immune system. Importantly, treatment with IL-15 and IL-15Rα restores the alcohol-mediated impairment of cNK development and maturation. This finding implies that the deleterious effects of alcohol might be traced to an impairment of upstream cells/pathways critical for the secretion of IL-15. For example, IL-15-producing CD11c^hi^ cells in the spleen are significantly decreased following chronic alcohol consumption. However, IL-15 can be produced from a variety of different cells including intestinal epithelial cells, thymic epithelial cells, keratinocytes, macrophages, and dendritic cells (80).

Mice depleted of NK cells by anti-AsGM1 antibody treatment displayed increased hepatic triglyceride levels and decreased serum alanine aminotransferase (ALT) levels following chronic ethanol exposure in mice, suggesting that NK cells mediate, in part, liver steatosis and injury. These data are also consistent with research that suggests that NK activation is beneficial in the short run, by increasing host defense against fibrosis and hepatic steatosis through selective cytotoxic activity. However, it is clear that chronic NK-cell activation contributes to liver damage (81). Cui et al. argued that the hepato-specific effects of NK cells were partially mediated by IFN-γ. IFN-γ downregulated the expression of several genes related to lipogenesis and fatty uptake including *Srebp-1*, *Fas*, *Acc*, *Gpat*, *Scd1*, and *Fat* (82). In addition, IFN-γ genetic knockout mice exhibited significantly more severe steatosis than WT mice. Finally, in recent work from our group we found that mice fed a binge-on-chronic ethanol diet exhibited reduced recruitment of NK cells and T cells to the lungs in response to bacterial pneumonia compared to control mice (83). Importantly, indole or probiotics supplementation restored pulmonary immune cell recruitment (NK cells and T cells) to the lungs of alcohol-fed mice and was dependent on AhR signaling, suggesting that alcohol-mediated intestinal dysbiosis and loss of specific microbial metabolites impairs recruitment of NK cells and T cells to the lungs to combat pathogenic insult (83). While NK-cell numbers and function are detrimentally affected by alcohol, it does not appear to affect the frequency of group I ILC.

In summary, alcohol arrests the development of NK cells in CD27^+^CD11b^+^ which could contribute to systemic dysregulation *via* interference with NK-driven IFN-γ signaling. Such dysregulation can contribute to the development of alcoholic liver disease, and studies on the depletion of cNK cells (*via* the anti-AsGM1 antibody) show increased steatohepatitis. Interestingly, NK-cell maturation can be rescued by the administration of IL-15 which suggests that IL-15 signaling is disrupted following alcohol administration. However, we have not rigorously identified which specific IL-15 producers are involved.

#### Group 2 ILCs

To our knowledge, there are no studies that have evaluated the effects of alcohol on ILC2 cells in any tissue. However, it is likely that ILC2s are affected by alcohol and contribute to alcohol-induced end-organ damage. ILC2 are critically important for type 2 inflammation and the regulation of normal host physiological responses, such as eosinophil and mast-cell recruitment, mucus accumulation, smooth-muscle hypercontractility, goblet-cell metaplasia, and the differentiation of macrophages toward an M2 phenotype. Alcohol is known to impair goblet-cell metaplasia and mucus accumulation (84–86), smooth-muscle hypercontractility (87), eosinophil and mast-cell recruitment (88–90), and alternative macrophage activation (91, 92). It follows that ILC2 dysregulation may contribute to alcohol-induced impairment of these processes. However, the role of ILC2 cells in these processes in the context of alcohol use is unknown and primed for future research.

#### Group 3 ILCs

While ILC3s have become a hot topic in immune research, little is known about the role of ILC3 in alcohol-induced end-organ damage. However, this is an ever-growing interest in the field. To date, only one study has examined the effects of alcohol on ILC3. Specifically, ethanol feeding was found to impair IL-22 production by ILC3s in the gastrointestinal tract (69). Loss of ILC3-mediated IL-22 production was driven by alcohol-associated dysbiosis and reduced levels of indole-3-acetic acid (I3AA). Noteworthily, supplementation of alcohol-fed mice with I3AA protected mice from steatohepatitis *via* increased expression of IL-22 and REG3G, as well as decreased bacterial translocation to the liver (69). Given that the importance of ILC3 in immune homeostasis is continually expanding, it is likely that ILC3 dysregulation may be a contributing factor during alcohol-induced end-organ damage. However, the role of ILC3 cells in the context of alcohol use is still understudied and primed for future research.

### Innate-like T lymphocytes

#### NKT and iNKT cells

The effects of alcohol on traditional NK (discussed above), NKT, and iNKT cells are the most well studied of effects on innate lymphocyte populations. However, given the ever-growing role and understanding of innate lymphocytes, even our knowledge of the effects of alcohol on these cell types is most likely in its infancy. Further, there are differences in the effects of acute and chronic alcohol consumption and there are likely subtle differences in the effects of alcohol across specific iNKT subsets. Broadly speaking, alcohol appears to increase immature iNKT-cell proliferation and maturation in the thymus with a corresponding increase in IFN-γ-producing iNKT-1 cells (65). *In vivo*, this facilitates a Th1-dominant immune response. This activation is interesting as it contrasts strongly to the inhibitory effects that alcohol exhibits on NK cells (discussed above).

Some have hypothesized that NK and iNKT cells may be interlinked through a system of contra-regulation (71). A significant fraction of iNKT cells produce interleukin-10 (IL-10). IL-10 is an interleukin known to antagonize the action of NK cells (72). For example, in contrast to NK-cell activity, iNKT cells promote hepatic steatosis by inhibiting the accumulation of NK cells and the release of IFN-γ (71). In addition, Jα18^-/-^ mice (a knockout model deficient in iNKT cells) demonstrated significantly higher levels of total NK-cell count and IFN-γ release following alcohol exposure, while WT mice exhibited a loss of total NK cells and IFN-γ. Likewise, iNKT-deficient Jα18^-/-^ mice appeared relatively protected from hepatic steatosis, but if these mice were also depleted of their NK cells by using the anti-AsGM1 antibody, alcoholic liver injury steatosis was significantly aggravated. Further, hepatic IL-10 was significantly upregulated, but no changes in TGF-β or IL-4 were noted. As noted above, iNKT cells are known for generating IL-10, which can inhibit NK activation and recruitment. In support of this cross talk, steatosis and liver damage were also alleviated in IL-10 KO mice, presumably *via* the suppression of NK cells.

In summary, alcohol enhances the development of iNKT cells, which promotes a Th1-dominant immune response. The extent to which the altered abundance of iNKT alters host health is unclear. However, dysregulation of iNKT may account for reports of alcohol-related signaling dysfunction involving IL-10 and other iNKT-derived cytokines. There is also evidence that increased iNKT activity promotes alcohol steatosis.

#### MAIT cells

Alcohol consumption influences MAIT-cell numbers and function through a variety of mechanisms. For example, chronic alcohol use is associated with impaired intestinal transport of riboflavin, as well as other B vitamins (93), which likely contributes to MAIT-cell depletion following chronic alcohol consumption. Work done by Zhang et al. demonstrates that there is a decrease in the abundance of MAIT cells in subjects with chronic alcoholic liver disease (78). Changes in MAIT-cell numbers also appear to depend on chronic ethanol consumption, as changes in MAIT-cell numbers were not observed following short-term binge drinking or short-term abstinence. Furthermore, the levels of peripheral MAIT cells were decreased and exhibited reduced antibacterial activity in subjects with alcoholic cirrhosis or severe alcoholic hepatitis (79). The hepatic expressions of the key transcription factors RORγt, PLZF, and Eomes were all reduced in subjects with severe alcoholic hepatitis (79).

Although alcohol can directly affect immune cells, it is worth noting that alcohol-related effects on intestinal bacterial antigens and/or metabolites, independent of ethanol, can deplete MAIT cells (79), which suggests that impairment in hepatic and circulating MAIT cells in patients with severe alcoholic hepatis is more likely due to chronic exposure to bacteria than to alcohol. Recent studies from our group have found that the number of MAIT cells in the mucosal tissues was significantly decreased in mice following binge-on-chronic alcohol feeding (47). However, CD69 expression was increased following alcohol feeding. Interestingly, the expression levels of Th1-specific cytokines and transcription factors were tissue specific. Th1-specific responses were decreased in the intestinal tract but enhanced in the lung and liver (47). Like previous studies which found a critical association of the gut microbiota with MAIT cells, we found that transplantation of the fecal microbiota from alcohol-fed mice into alcohol-naïve mice resulted in a MAIT-cell profile similar to those seen in our alcohol-feeding model (47). Importantly, the differences observed between MAIT cells from alcohol- and control-fed mice were mitigated by antibiotic treatment. Further, in subjects with alcohol-associated liver disease, as well as in rodent ethanol-feeding models, there is increased intestinal permeability with systemic distribution of bacterial products, such as LPS and bacterially derived riboflavin (94, 95). Riboflavin is known to activate MAIT cells, at least in the short term; however, long-term exposure may lead to MAIT-cell exhaustion, which has been reported in chronic conditions like HIV (96, 97).

In summary, alcohol decreases the function and abundance of MAIT cells. However, these deficiencies are not caused solely by the direct effects of alcohol and its metabolites on the eukaryotic cells of the host. Rather, it appears that alcohol-related changes to the microbiota can produce MAIT-cell dysfunction independent and in addition to changes caused directly by alcohol.

#### γδ T cells

Like ILC3 cells, there is a paucity of data regarding the effects of alcohol on γδ T cells. Currently, the effects of alcohol on dermal immunological responses, particularly γδ T cells, are the most well characterized. In a murine model, chronic EtOH feeding leads to a loss of specific subsets of dermal T cells, including Foxp3+ regulatory T cells and both CD3^hi^Vγ3+ and CD3^int^Vγ3-γδ T cells (73). EtOH was also correlated with an impaired functional capacity of dermal γδ T cells (the prototypical dermal cells that produce IL-17). Precisely, IL-17 production following anti-CD3 stimulation was significantly reduced in dermal γδ T cells (73). Further, lymph node-associated γδ T cells isolated from EtOH-fed mice also exhibited diminished IL-17 production following stimulation (73). In similar studies, hepatic IL-17A production was found to be cell type specific depending on alcohol exposure. In alcohol-naïve mice, IL-17 is produced primarily by hepatic γδ T cells. However, following acute-on-chronic EtOH consumption, the secretion of IL-17A was shifted to a more CD4^+^ T-cell mediated response (74). Noteworthily, these results were not seen in TLR3 KO or Kupffer cell-depleted mice, which suggest that TLR3 activation in Kupffer cells leads to an elevated IL-1β expression, thus driving IL-17A secretion by γδ T cells early during alcohol-associated liver disease and increased CD4+ T-cell secretion of IL-17A during the end stage of alcohol-associated liver disease (74).

## Discussion

Alcohol is known to impair immune function and perturb immune homeostasis. It follows that organ systems which rely on immune signaling for proper functioning are also impaired. Some systems, like the nervous system may be altered in subtle ways that alter behavior (75). In contrast, systems which directly encounter pathogens from the environment can become more susceptible to infection and injury (76, 77). However, our understanding of the mechanisms by which this occurs remains in its infancy. Multiple researchers have reported that alcohol use can deplete critical cell subpopulations, by impairing cell maturation and chemotaxis. The depletion of these cells can propagate multiple deleterious effects. For example, a depletion of NK cells (cytotoxic, IFN-γ secreting) would be expected to impair immune responses reliant on cytotoxicity; however, NK depletion would also be expected to impact tissue IFN-γ levels and thereby attenuate responses by the adaptive and innate arms of the immune system. Examples of cell depletion and signaling disruption have been reported for many types of innate immune cells. However, it is also worth recognizing that alcohol and alcohol-related metabolites can interact with a variety of lymphocytes in nuanced ways through mechanisms other than cellular depletion. Some cell types, such as MAIT cells, may be mediated through indirect pathways that involve the microbiota. This review was primarily concerned with innate-like T lymphocytes, and therefore, we emphasized examples like the observation that alcohol increases iNKT IL-10 secretion. Consider however that IL-10 signaling is also heavily utilized by innate-like B cells, a group important for IgM and as the first line of defense against infection (98). It would be worth exploring the effects of alcohol on these cells, both in their secretion of IL-10 and in their ability to repel infection. At the time of this review, we found little research discussing the effects of alcohol on innate-like B cells. Overall, research on the effect of alcohol on all innate-like lymphocytes remains underdeveloped.

## Author contributions

DV, KR-C, and DS reviewed the literature and wrote the manuscript. All authors contributed to the article and approved the submitted version.

## Funding

The work was supported by the National Institute on Alcohol Abuse and Alcoholism Grants: #K99-AA026336 and #R00-AA026336. The content is solely the responsibility of the authors and does not necessarily represent the official views of the National Institutes of Health. The funders had no role in study design, data collection and analysis, decision to publish, or preparation of manuscript.

## Conflict of interest

The authors declare that the research was conducted in the absence of any commercial or financial relationships that could be construed as a potential conflict of interest.

## Publisher’s note

All claims expressed in this article are solely those of the authors and do not necessarily represent those of their affiliated organizations, or those of the publisher, the editors and the reviewers. Any product that may be evaluated in this article, or claim that may be made by its manufacturer, is not guaranteed or endorsed by the publisher.
